# Trace Levels of Staphylococcal Enterotoxin Bioactivity Are Concealed in a Mucosal Niche during Pulmonary Inflammation

**DOI:** 10.1371/journal.pone.0141548

**Published:** 2015-10-28

**Authors:** Antoine Ménoret, Julia Svedova, Bharat Behl, Anthony T. Vella

**Affiliations:** Department of Immunology MC3710. University of Connecticut Health, 263 Farmington Avenue, Farmington, CT 06032, United States of America; Instituto Butantan, BRAZIL

## Abstract

Pathogen and cellular by-products released during infection or trauma are critical for initiating mucosal inflammation. The localization of these factors, their bioactivity and natural countermeasures remain unclear. This concept was studied in mice undergoing pulmonary inflammation after Staphylococcal enterotoxin A (SEA) inhalation. Highly purified bronchoalveolar lavage fluid (BALF) fractions obtained by sequential chromatography were screened for bioactivity and subjected to mass spectrometry. The Inflammatory and inhibitory potentials of the identified proteins were measured using T cells assays. A potent pro-inflammatory factor was detected in BALF, and we hypothesized SEA could be recovered with its biological activity. Highly purified BALF fractions with bioactivity were subjected to mass spectrometry. SEA was the only identified protein with known inflammatory potential, and unexpectedly, it co-purified with immunosuppressive proteins. Among them was lactoferrin, which inhibited SEA and anti-CD3/-CD28 stimulation by promoting T cell death and reducing TNF synthesis. Higher doses of lactoferrin were required to inhibit effector compared to resting T cells. Inhibition relied on the continual presence of lactoferrin rather than a programming event. The data show a fraction of bioactive SEA resided in a mucosal niche within BALF even after the initiation of inflammation. These results may have clinical value in human diagnostic since traces levels of SEA can be detected using a sensitive bioassay, and may help pinpoint potential mediators of lung inflammation when molecular approaches fail.

## Introduction

There is increased recognition of *S*. *aureus* infection in the respiratory tract of patients not only as a single pathogen but also as a pivotal co-infection agent especially in combination with viruses such as influenza [1,2]. Diagnosing the root pathogenic mechanism of co-infection is challenging since complex biological processes of different infectious agents along with the host are involved. Nevertheless, there is often an ignored role for pathogen-generated toxins that can be secreted during infection. An excellent example are the enterotoxins released by *S*. *aureus* that have classically been grouped as superantigens [3]. Thus, besides the infectious agents themselves the presence of toxins such as Staphylococcal enterotoxins (SE) provides another layer of complex pathogenicity that directly impacts adaptive immunity.

Perhaps the greatest threat of SE are their potential to induce severe or even lethal shock as observed in classical cases of Toxic Shock Syndrome [4], but also documented in less well publicized instances of shock after endonasal surgery [5]. In either situation disease onset is rapid, and largely manifested by a massive cytokine storm triggered by SE-activated T cells. This stems from the oligoclonal activation of T cells by SE, which relies not on processed antigen from the pathogen but by bridging the MHC II molecule to a specific TCR Vβchain [3]. Not only do CD4 T cells become activated but CD8 T cells are also potently stimulated leading to substantial clonal expansion, effector differentiation and systemic migration throughout the body [6]. Thus, it is not surprising that SE mediate pathogenic outcomes in lung as observed in several pulmonary inflammation models of asthma and acute lung injury [7,6,8]. Nevertheless, there is growing evidence that SE may be involved in human pulmonary maladies including rhinosinitus [9] and as a co-morbid of asthma [10]. Recent evidence has also implicated the presence of SE in victims of sudden infant death syndrome [11]. Taken together, the diagnosis and rapid implementation of countermeasures against SE might aid in treating and understanding T cell associated severe pulmonary diseases including status asthmaticus and others [12,10,13].

A difficulty in determining a role for SE in human disease is that molecular diagnostics can only locate the presence of a factor after reaching a certain concentration threshold. An even more difficult challenge is that sensitive PCR diagnostics for the bacteria are not very useful in the detection of SE since these proteins often migrate from the sites of colonization [14]. For example, SE in lung mucosa may be localized away from the colonizing bacteria in the nasal polyps, making a PCR-based diagnosis of a lung specimen inconclusive. One might also consider TCR Vβ expansion as an indicator of SE, but this would not infer imminent presence and is also complicated by the loss of polyclonality of TCRs in CD8 T cells of people especially the elderly [15,16]. Also, SE by virtue of the fact that they are Proteinaceous, effective at very low concentrations, and have a high binding avidity for MHC II, makes them difficult to detect in patient samples. Thus, a major gap is how SE can be efficiently detected, but perhaps even more importantly tracking SE bioactivity which an ELISA or immunoblot cannot.

To address this idea we set out to determine if SEA could be recovered and if it retained biological activity. We tested this idea using a bioassay where cells produce IFN-γ in response to LPS or cytokines and found that BALF from intranasally-SEA treated mice contain a potent capacity to stimulate IFN-γ release. Confirming our hypothesis, we found trace levels of SEA in fractionated BALF even after inflammation was initiated. Purification of SEA from BALF showed that its presence was undetectable by immunoblot and that a T cell dependent bioactivity assay was necessary for its identification; perhaps even moreso than mass spectrometry. Secondly, SEA co-purified with host lactoferrin in fractionated BALF, which weakened T cell IL-2 and TNF synthesis. Specifically, cytokine responses were inhibited only when lactoferrin was in the continual presence of the cells. Lactoferrin did not pre-program protective or attenuated responses, instead its presence was a requirement especially for effector T cells. These results suggest that bioactive SE may be present in trace amounts in BALF of patients with severe respiratory events. A sensitive bioactivity assay may assist in identifying disease-mediating agents in complex cases of rapid shock seen in intensive care units.

## Materials and Methods

### Mice and immunization

C57BL/6 and TCR β/γ mice were purchased from the National Cancer Institute (Frederick, MD) or the Jackson Laboratory (Bar Harbor, ME). All mice were maintained in the central animal facility at the University of Connecticut Health (UCH) in accordance with federal guidelines. The present study was approved by the University of Connecticut Health’s Animal Care Committee. Mice were anesthetized with isoflurane (Vedco Inc., Saint Joseph, MO) and given 1 μg of SEA diluted in 50 μl of BSS, or BSS alone by intranasal (i.n.) route as previously described [17].

### Reagents, antibodies and ELISA

Staphylococcus enterotoxin A (SEA) were purchased from Toxin Technology Inc. (Sarasota, FL). Lipopolysaccharide (LPS) from *salmonella enteric typhimurium* was purchased from Sigma (St. Louis, MO). Mouse recombinant IL-6, IL-12, IL-18, IL-33, IL-36β, IL-36β, and TNF were purchased from R&D Technologies (Kingstown, RI). Mouse recombinant IL-1α and IL-1β were purchased from BD Biosciences (San Jose, CA). IFN-α was purchased from PBL Assay Science (Piscataway, NY). Bovine serum albumin (BSA), was purchased from Gemini Bio-Products (West Sacramento, CA) and bovine lactoferrin and transferrin were purchased from Sigma (St. Louis, MO). Proteinase K and ionomycin were purchased from Life Technology (Grand Island, NY). Phorbol 12-Myristate 13-Acetate (PMA) and Brefeldin A (BFA) was purchased from EMD Millipore Corporation (Billerica, MA). Anti-Lactoferrin rabbit polyclonal antibody (ab135710) was purchased from Abcam (Cambridge, MA), anti-SEA antibody (GTX39112) was purchased from GeneTex and biotinylated using EZ-Link Sulfo-NHS-Biotin biotinylation kit from Thermo Scientific (Asheville, NC). Anti-mouse and anti-rabbit IgG secondary HRP-conjugated antibodies were purchased from Santa Cruz Biotechnology (Santa Cruz, CA). Avidin-HRP was purchased from BIORAD (Hercules, CA). Anti-CD4-V450, -TCR Vβ3-PE and -TNF-AF700 were purchased from BD biosciences (San Jose, CA). IFN-γ, IL-6, TNF and IL-2 ELISA kits were purchased from BD biosciences Pharmingen (Mountain View, CA). Mouse serum amyloid A (SAA) ELISA kit was purchased from Immunology Consultants Laboratory, Inc. (Portland, OR).

### BALF processing and bioassay

**Fig 1:**
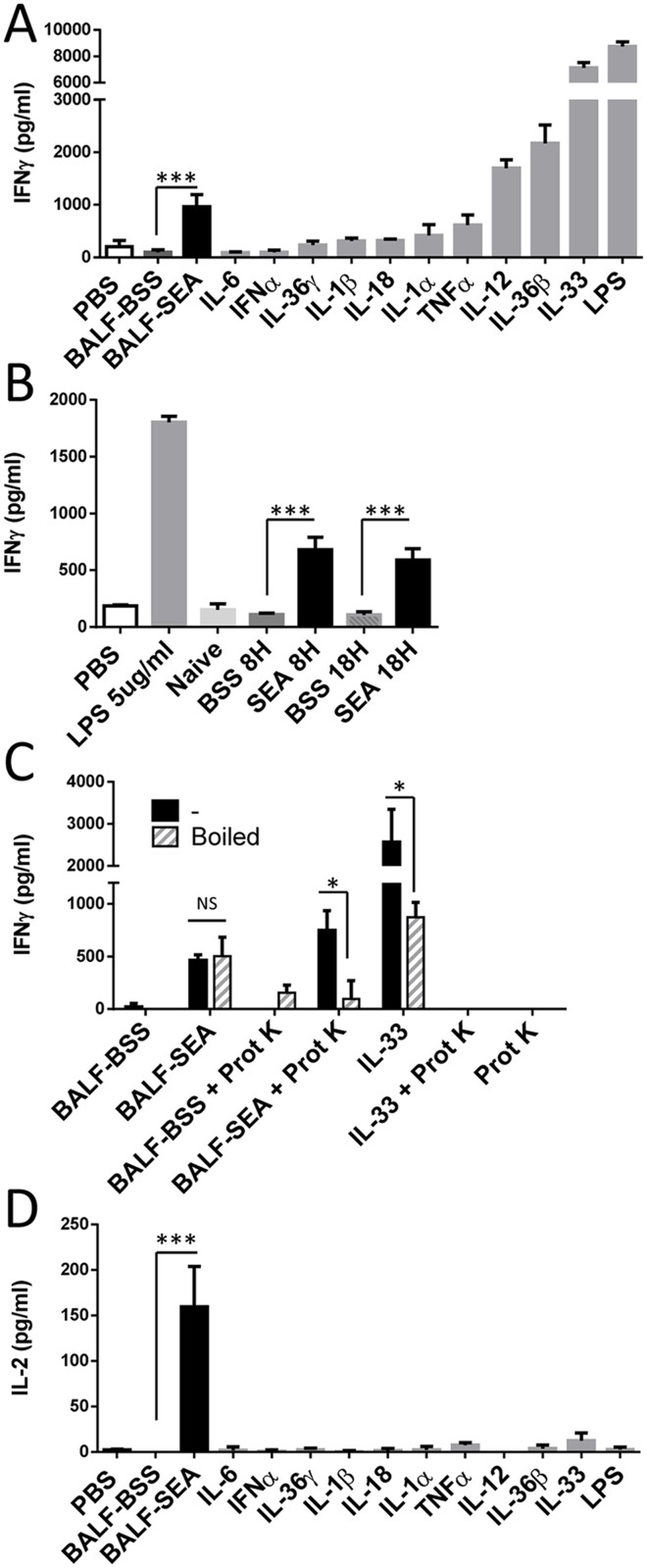
BALF of mice treated with SEA contain a heat resistant proteinaceous pro-inflammatory factor. (**A**) BALF obtained from mice 16 h after i.n. SEA (1 μg) diluted in BSS (BALF-SEA) or BSS alone (BALF-BSS) were tested in a bioassay as described in material and method section. Briefly, mouse splenocytes were pretreated with IL-12 (2 ng/ml) for 16–18 h, washed and rested for 5 h. Primed splenocytes (200,000/well) were incubated with BALF and controls. Culture supernatants were obtained after 16–18 h and assayed for IFN-γ by ELISA. For comparison, proinflammatory cytokines at a final concentration of 5 ng/ml, except IFN-α (1000 U/ml), and LPS (5 μg/ml) were added to the bioassay. Bar graphs show IFN-γ secretion measured in triplicate. Representative of 1 out 3 experiments is shown. (**B**) BALFs obtained 8 h and 18 h after i.n. SEA and BSS were compared in a bioassay. Bar graphs show IFN-γ secretion measured in triplicate. Representative of 1 out 4 experiments is shown. (**C**) BALFs and IL-33 (bioactive protein control) incubated with and without proteinase K at 37°C for 18 h, were either boiled for 10 min or left untreated and tested in a bioassay as described above. Bar graphs show IFN-γ secretion measured in triplicate. Representative of one out three experiments is shown. (**D**) Culture supernatants obtained with BALFs, cytokines and LPS applied to a bioassay as described in **A** were tested for IL-2. Bar graphs show IL-2 secretion measured in triplicate. Representative of 1 out 3 experiments is shown. For all panels statistical significance between groups was evaluated by two-tailed Student’s *t* tests. The error bars indicate the standard error of the mean between triplicates. * p<0.05, *** p<0.001.

**Fig 2:**
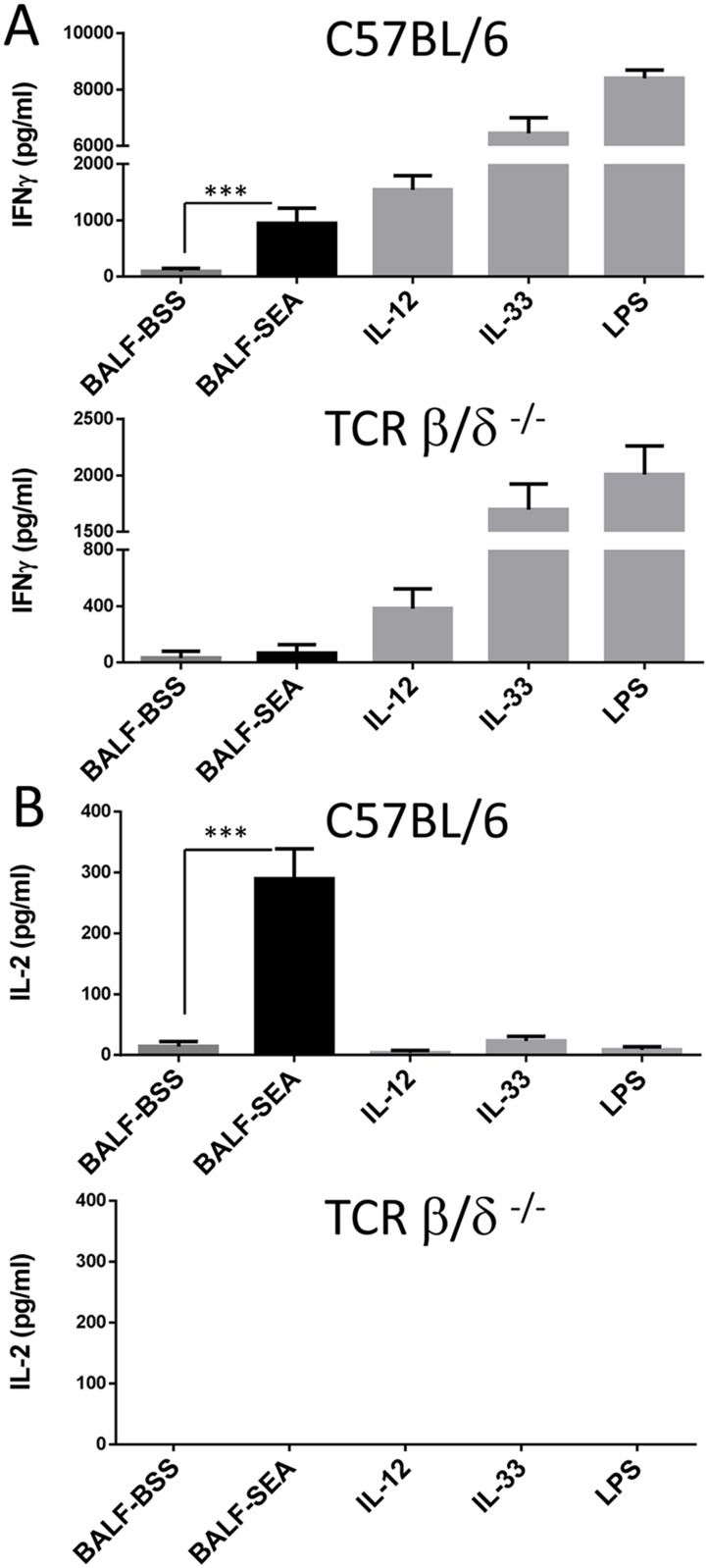
The inflammatory activity induced by BALF-SEA is T cell dependent. BALFs obtained from mice 16 h after i.n. SEA and BSS were tested in a bioassay using C57BL/6 splenocytes (top panel) and TCR β/γ^-/-^ splenocytes (bottom panel) and supernatants were assayed for IFN-γ (**A**) and IL-2 (**B**). Bar graphs show cytokines secretion measured in triplicate with n = 3. The data are representative of 3 independent experiments. Statistical significance between BALF-BSS and BALF-SEA was evaluated by two-tailed Student’s *t* tests. The error bars indicate the standard error of the mean between triplicates.

BAL was collected by lavage of mouse lung with sterile PBS. BAL was centrifuged at 1,000 rpm at 4°C to separate cells from BALF and 0.2 micron filtered as described earlier [18]. The immunostimulatory activity contained in the BALF was measured using a bioassay adapted from a technique used to measure IL-18 bioactivity [19]. Briefly, RBC depleted mouse splenocytes were incubated overnight with IL-12 (2 ng/ml) in Figs 1 and 2 only, washed and incubated in tissue culture medium without IL-12. After 5 h, splenocytes were seeded in 96-well flat-bottom plates (2 x 10^6^ per well in triplicate) and stimulated with 20 μlBALF from SEA or vehicle (alone) exposed mice for 16–18 h. Supernatants were measured for IL-2 and IFN-γ levels by ELISA.

### Chromatography

BALF samples were separated by size exclusion chromatography using Sephacryl 100-HR (Sigma, St Louis, MO) equilibrated in sterile PBS and run at 4°C at 0.5 ml/min. Absorption of the proteins was recorded at 280 nm and fractions collected every 1.5 ml. Anion exchange chromatography was performed using High Q (BIORAD, Hercules, CA) equilibrated with 20 mM phosphate (pH 7.0). The samples were applied to the column, washed at 1 ml/min and eluted with a linear gradient of 20 mM phosphate, 1 M NaCl (pH 7.0). Protein detection was recorded at 280 nm and fractions collected every 1 ml. Cation exchange chromatography was performed using High S (BIORAD, Hercules, CA) equilibrated with 20 mM MES (pH 5.5). The samples were applied to the column, washed at 1.5 ml/min, eluted with a linear gradient of 20 MES (pH 5.5) 1 M NaCl and fractions collected every 1 ml. BALF was also fractionated directly by cation exchange chromatography using the same protocol as described above except the elution gradient was performed with 20 MES (pH 5.5), 2 M NaCl. All buffers were filtered at 0.2 microns.

### Mass Spectrometry

The fractions of interest were lyophilized, resuspended in trypsin digestion buffer, peptides were obtained and identified by LC MS/MS on an Orbitrap ELITE (Yale Keck facility, New Heaven, CT) using the following parameters: maximum number of missed cleavage: 1; partial oxidation of met: allow; enzyme: trypsin; monoisotopic mass threshold: 10 PPM; fragment mass tolerance 0.5 Da; monoisotopic or average: monoisotopic. All MS/MS samples were analyzed using Mascot (Matrix Science, London, UK; version Mascot). Mascot searched the SWISSPROT database for the mouse genome (*Mus musculus)* assuming the digestion by trypsin and was further search for the sequence of SEA (*Staphylococcus aureus* enterotoxin A).

### SDS-PAGE and immunoblotting

BALF and proteins were resuspended in denaturing SDS sample buffer and resolved by SDS-PAGE and immunoblotted as described earlier [20]. Briefly, samples were resolved on 4–15% SDS PAGE, transferred onto 0.2 μm nitrocellulose membrane (BIORAD, Hercules, CA), probed with primary antibodies, washed, then incubated with secondary HRP-conjugated antibodies or avidin-HRP. Western blot detection was performed using ECL plus (Amersham, Arlington Heights, IL).

### Flow cytometry

Following previous procedures [20], RBC-depleted spleens and lymph node cells were resuspended with staining buffer (BSS, 3% FBS and 0.1% sodium azide), followed by blocking with a mixture of normal mouse serum, anti-Fc receptor supernatant from the 2.4 G2 hybridoma [21], and human gamma globulin. Incubation with labeled primary antibodies and flow cytometry analysis was carried out as before [20]. For intracellular cytokine staining, spleen cells were cultured for 4 h with SEA or PMA + Ionomycin in the presence of brefeldin A (BFA), and then stained for CD4, Vβ3 and TNF as described before [20]. Cell viability was assessed using MitoFlow kit purchased from Cell Technology (Fremont, CA) as before [22].

### Statistics

Mean, standard error of the mean and t test were calculated using GraphPad Prism6 software (La Jolla, CA).

## Results

### BALF of mice treated with SEA contain a stable T cell dependent pro-inflammatory factor

**Fig 3:**
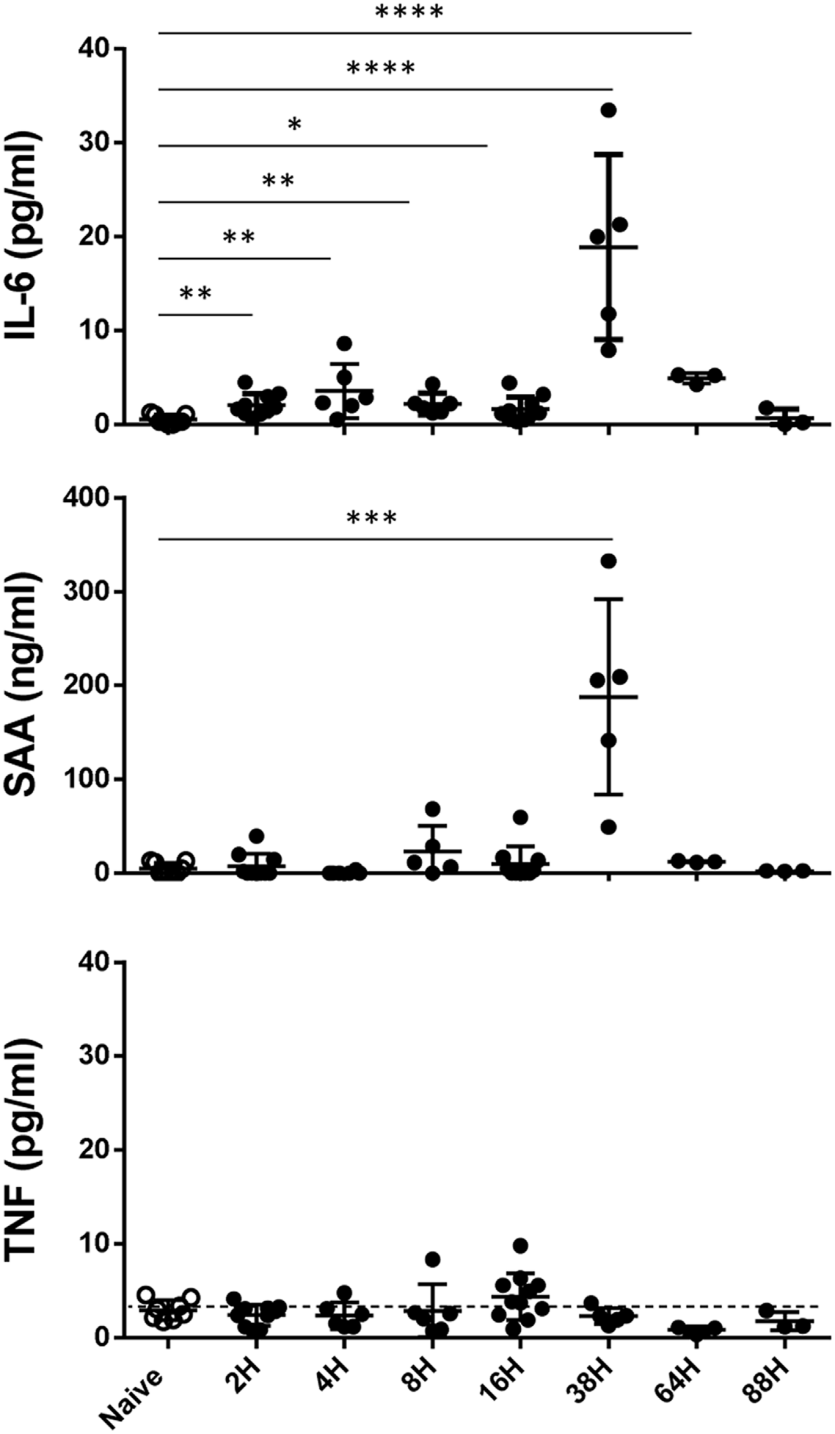
SEA induces classical pro-inflammatory factors in BALF. BALF obtained 2–88 h after i.n. SEA inhalation was tested for the presence of IL-6, TNF and SAA by ELISA. Plots show cytokine secretion, each symbol represents one mouse. BALF were collected from 10 experiments. The error bars indicate the standard error of the mean between the mice in one group. Statistical significance between the groups was evaluated by two-tailed Student’s *t* tests.

Our previous data demonstrated that SEA inhalation induced significant pulmonary inflammation coincident with the appearance of cell damage suggesting the presence of DAMPs, alarmins and/or cytokines [18]. To study this possibility, BALF from intranasal SEA-treated mice was used to induce IFN-γ release using a sensitive cell bioassay relying on IL-12-preconditioning of spleen cells [19]. BALF from SEA-treated mice (BALF-SEA) contained an activity that can also be induced by factors such as IL-12, IL-36β, and especially IL-33 (Fig 1a) an alarmin involved in SEA mediated lung inflammation [23]. This activity occurred as early as 8 h after SEA treatment, was present at 18 h, and required SEA since vehicle (BALF-BSS) was ineffective (Fig 1b). The activity was proteinase K resistant unless boiled for 10 minutes, suggesting a heat resistant protein, in contrast to IL-33 that was heat and proteinase K sensitive (Fig 1c). Like LPS, BALF-SEA stimulated IFNγ, but this did not rule out a role for adaptive immune cell cytokine production. As expected, neither LPS nor any innate cytokine induced IL-2, but BALF-SEA did (Fig 1d). To test if cytokines produced in the bioassay were derived from innate or adaptive immune cells, IFN-γ and IL-2 were measured using WT versus TCR β/γ mice spleen cells. As expected, IFN-γ was induced by BALF-SEA, IL-12, IL-33 and LPS (Fig 2a, top panel), but BALF-SEA did not induce IFN-γ in the absence of T cells even though the innate factors were able to do so (Fig 2a, bottom panel). Similarly, IL-2 was only induced by BALF-SEA when T cells were present (Fig 2b). Moreover, we looked for classical inflammatory factors secreted in the BALF after SEA inhalation and detected IL-6, serum amyloid A (SAA) but no TNF (Fig 3). IL-6 and SAA were detected in the BALF latter (38h) than the pro-inflammatory activity described in Figs 1 and 2 suggesting the presence of an earlier factor.

### Purification and identification of the pro-inflammatory activity present in BALF-SEA

**Fig 4:**
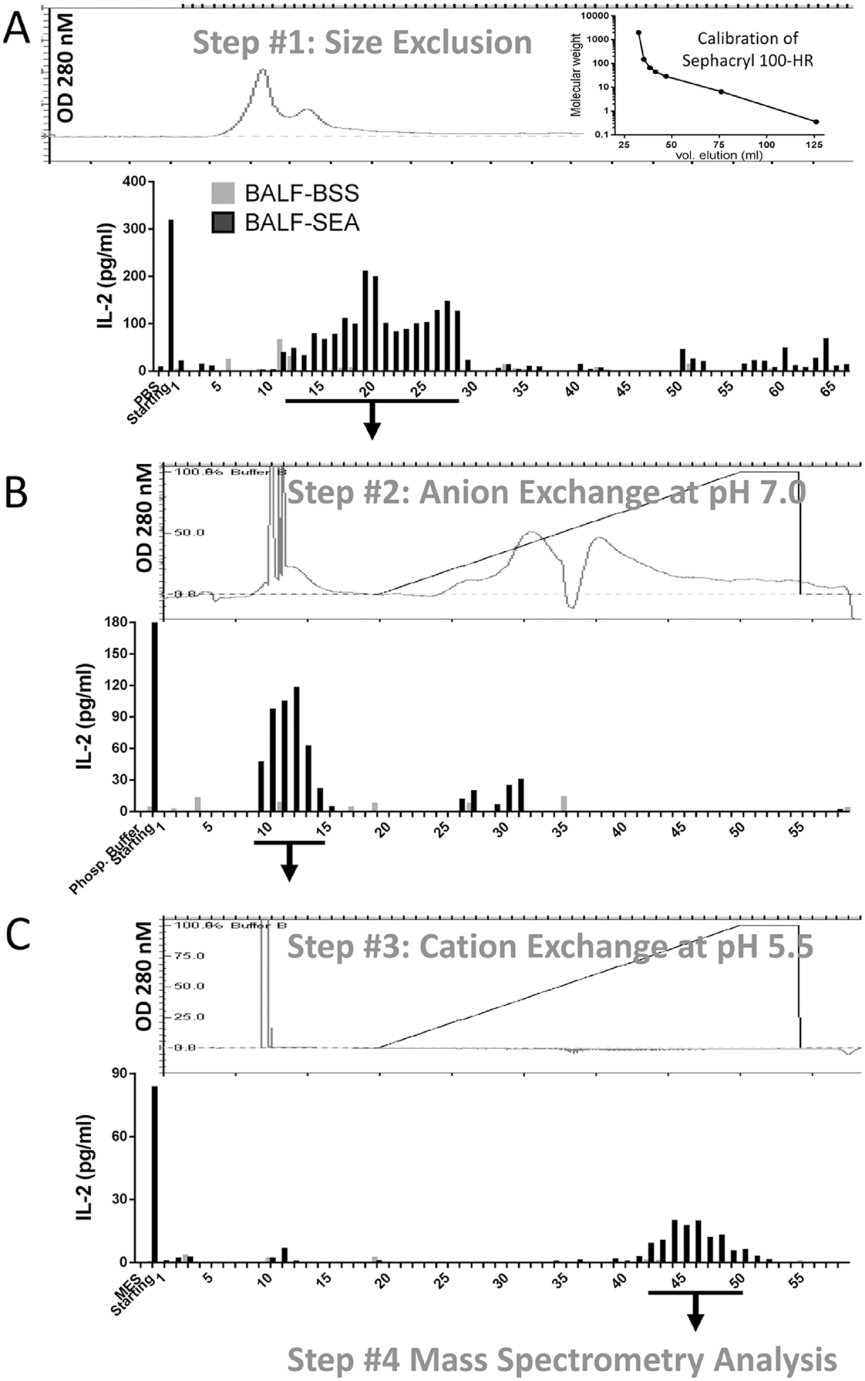
Chromatographic purification of the pro-inflammatory activity present in BALF-SEA. (**A**) BALFs were obtained from mice 16 h after i.n. SEA or BSS, concentrated using a 3 kDa centricon and loaded on a size exclusion column (Sephacryl 100-HR). Protein detection was recorded at 280 nm (top panel) and fractions from BALF-SEA and BALF-BSS were tested in the bioassay (bottom panel). BALF-SEA fractions with bioactivity were pooled and concentrated on centricon 3 kDa while the corresponding fractions from BALF-BSS were processed identically. Each sample run was a pool of BALF obtained from 3–5 mice. Representative of 1 out 5 experiments is shown. (**B**) Samples were concentrated, the buffer was exchanged on a PD-10 column to 20 mM Phosphate pH 7.0, applied to an anion exchange column (High Q) and an elution profile was developed with a linear gradient of 0–1 M NaCl. Protein detection was recorded at 280 nm (top panel), bound and unbound fractions were collected, tested in a bioassay (bottom panel), and pooled as described in **A**. Representative of 1 out 4 experiments is shown. (**C**) Samples were concentrated, buffer exchanged on a PD-10 column to 20 mM MES pH 5.5, applied to a cation exchange column (High S) and an elution profile was developed with a linear gradient of 0–1 M NaCl. Protein detection was recorded (top panel), bound and unbound fractions collected, tested (bottom panel) and pooled as described in **A**. Representative of 1 out 3 experiments is shown.

We hypothesized that SEA was in the BALF and responsible for this activity. To test this idea an unbiased chromatographic strategy to isolate the factor was performed using the IL-2 bioassay. A size exclusion chromatography approach was used to generate fractions containing IL-2 inducing activity that eluted as a broad peak between 15 and 45 kDa, which was based on the calibration curve (Fig 4a-inset). The fractions with the IL-2 inducing activity from BALF-SEA were pooled, concentrated, and applied for a second step of purification using anion exchange. The corresponding fractions from BALF-BSS were processed identically. Most of the proteins bound the anion exchange column and eluted in the salt gradient (Fig 4b, top panel). In contrast, the majority of the IL-2 inducing activity was detected in the unbound material (Fig 4b, bottom panel), consistent with SEA’s isoelectric point of 7.7. Fractions with the IL-2 inducing activity from Fig 4b were pooled, concentrated and then placed on cation exchange at pH 5.5 (Fig 4c, top). No protein was detected in the salt elution gradient but the IL-2 inducing activity was detected (Fig 4c, bottom). After mass spectrometry of the pooled IL-2 inducing fractions SEA was indeed recovered 16 h post inhalation at trace amounts based on percentage of peptide coverage that was only 9.3% (Table 1).

**Table 1:** Proteins identified by Mass Spectrometry.

**PROTEINS DETECTED ONLY IN BALF-SEA**			
**Protein Name**	% coverage *	High identity Peptides **	Low identity Peptide ***
***Lactoferrin***	**32.5**	***8***	***7***
***Staphylococcus Enterotoxin A (SEA)*, *S*. *aureus***	**9.3**	***2***	***0***
Lysozyme C-1	8.1	1	0
Ubiquitin-40S ribosomal protein S27a	16	1	0
Collagen alpha-1(VII) chain	0.7	1	1
Annexin A2	2.9	1	0
Ankyrin repeat domain-containing protein SOWAHC	1.8	1	0
Estradiol 17-beta-dehydrogenase 8	10.4	2	0
E3 ubiquitin-protein ligase NEURL1B	1.5	1	1
**PROTEINS DETECTED IN BOTH BALF-SEA AND BALF-BSS**			
**Protein Name**	% coverage *	High identity Peptides **	Low identity Peptide ***
***Transferrin***	**50.9**	***28***	***6***
Hemoglobin subunit beta-1	28.6	4	0
Superoxide dismutase [Cu-Zn]	72.7	5	2
Hemoglobin subunit alpha	33.1	4	1
Serum albumin	8.9	3	2
Protein S100-A9	12.4	2	0
Transthyretin	25.9	2	1
Solute carrier family 26 member	5.8	2	0
Arf-GAP with SH3 domain	0.8	1	0

Pooled cation exchange chromatography fractions (Fig 4c) from BALF-SEA and corresponding BALF-BSS fractions were lyophilized, and identified as described in Material and Method section.

*Percentage of the entire protein sequence that is covered by the sequences of the identified peptides.

**Peptides identified with score greater than identity score.

***Peptides identified with score between homology score and identity score.

### SEA co-migrates with immunosuppressive proteins

**Fig 5:**
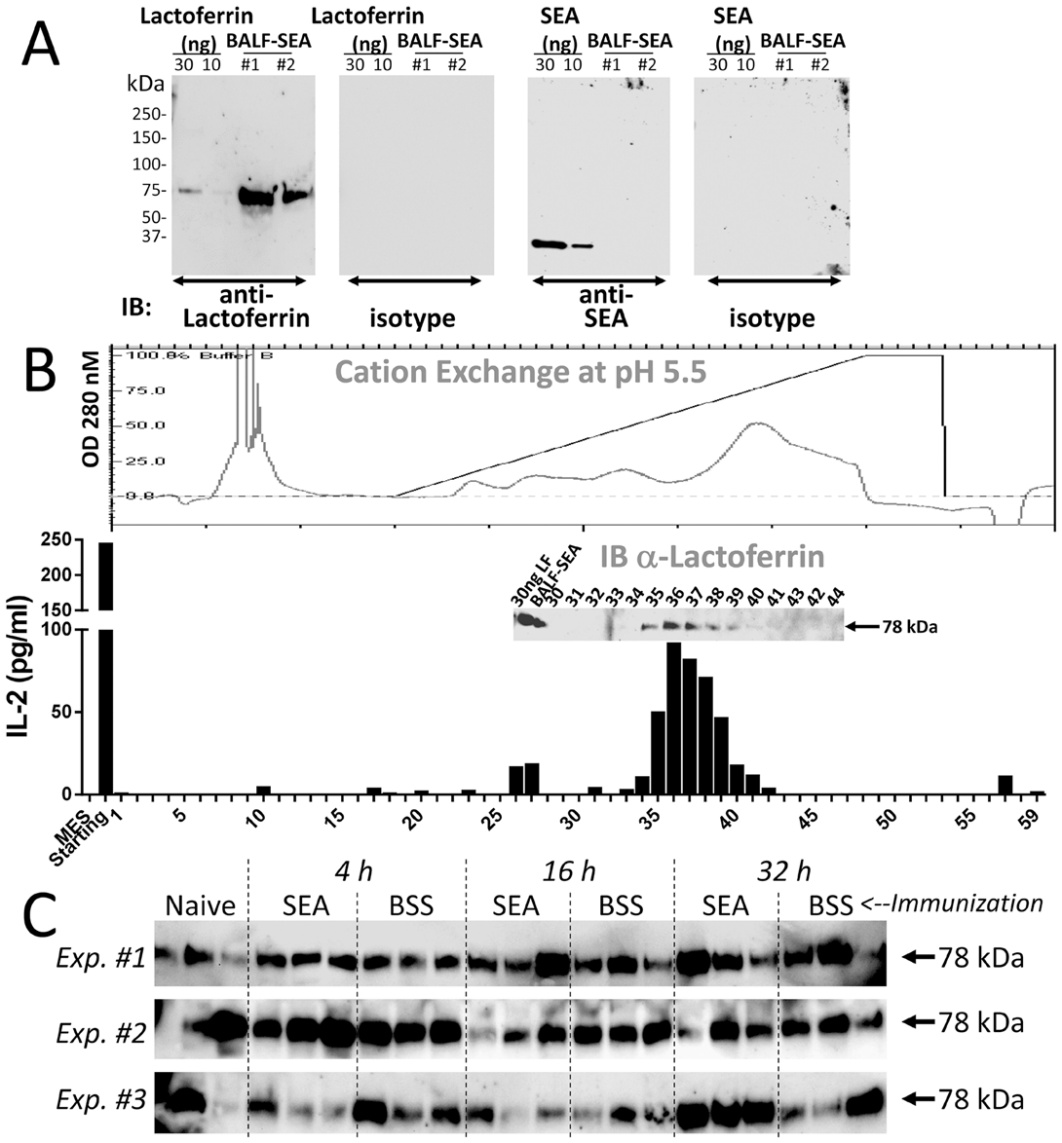
Lactoferrin co-migrates with SEA. (**A**) BALF-SEA was resolved by 4–15% gradient SDS-PAGE under reducing and denaturing conditions, transferred to nitrocellulose membrane and probed with anti-lactoferrin and anti-SEA antibodies as described in the Materials and Methods. Purified Lactoferrin and SEA (30 and 10 ng) were loaded in parallel to serve as positive controls. Specificity of the antibody was assessed using isotype control. The data are representative of 2 (lactoferrin) and 3 (SEA) independent experiments. (**B**) 16 h BALF-SEA was directly fractionated by cation exchange chromatography. Fractions were tested in a bioassay and immunoblotted for lactoferrin (inset). Chromatogram of protein absorption at 280nM (top panel) and bar graphs of IL-2 secretion (bottom panel) are shown. Representative of 1 out 3 experiments is shown. (**C**) BALF from naïve mice, and BALF from mice having received a time course of i.n. SEA and BSS were immunoblotted with anti-lactoferrin antibodies and 3 experiments are shown.

Lactoferrin was also detected in BALF-SEA which seemed to co-migrate with SEA since it was not detected by mass spectrometry in BALF-BSS (Table 1). It has been shown previously that lactoferrin can impede inflammation [24], attenuate SEB activity [25] and therefore we hypothesized it could also affect SEA activity. Immunoblotting showed that lactoferrin was present at high levels in BALF-SEA while SEA was undetectable (Fig 5a). To test if in BALF-SEA lactoferrin and SEA co-migrated, cation exchange chromatography was used and elution occurred between 1.03 M and 1.3 M NaCl (Fig 5b, top panel). Specifically, IL-2-inducing activity was found precisely where lactoferrin was detected (Fig 5b, bottom panel). Thus, lactoferrin may function as an innate factor. Lactoferrin was present in BALF-SEA and -BSS through a time course, and also detectable in BALF from naïve mice (Fig 5c). Thus, SEA fractionates at least with a portion or subspecies of lactoferrin. In sum, lactoferrin is in BALF-BSS and BALF-SEA before protein fractionation, but after fractionation a sub fraction of lactoferrin co-migrates with SEA from BALF-SEA but not in the equivalent fraction from BALF-BSS.

**Fig 6:**
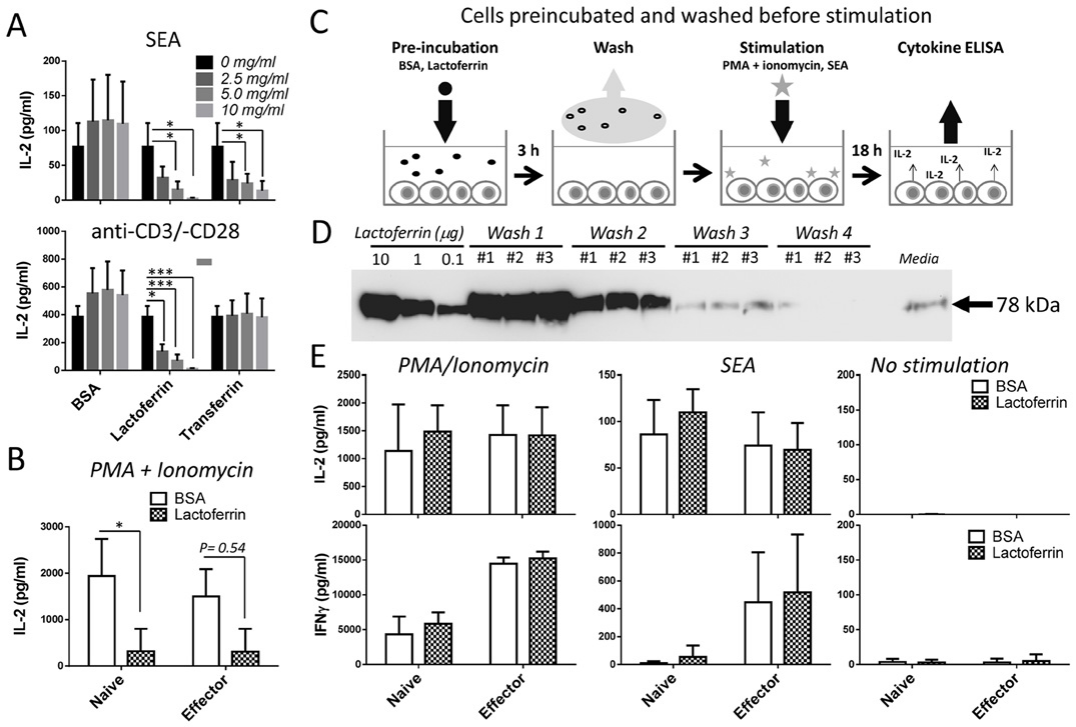
Lactoferrin inhibits IL-2 secretion. (**A**) Mouse splenocytes stimulated with SEA (10 ng/ml) (top panel) and soluble anti-CD3/-CD28 (each 1 μg/ml) (bottom panel) were co-incubated with increasing doses of BSA (negative control), lactoferrin and transferrin. Culture supernatants were assayed for IL-2 after 18 h and the bar graphs show IL-2 secretion. Data are representative of the average of 3 independent experiments with n = 3. The error bars indicate the standard error of the mean between 3 biological replicates. Statistical significance between treatments was evaluated by two-tailed Student’s *t* tests. * p<0.05, ** p<0.01, *** p<0.001. (**B**) Mouse splenocytes from either unimmunized mice (*Naïve*) or 3 day post immunization with 0.5 μg SEA and 10 μg LPS (*Effector*) were incubated with lactoferrin (white bars) or BSA (grey bars) (5 mg/ml) at 37°C with PMA (50 ng/ml) + Ionomycin (1 μg/ml). Culture supernatants were assayed for IL-2 after 18 h and the data are representative of the average of 3 independent experiments with n = 3. The error bars indicate the standard error of the mean between 3 biological replicates. Statistical significance between lactoferrin and BSA was evaluated by two-tailed Student’s *t* tests. (**C**) Cells were incubated with lactoferrin or BSA (5 mg/ml) for 3 h at 37°C, washed 4 times with tissue culture medium, and then stimulated with SEA (10 ng/ml) or PMA (50 ng/ml) + Ionomycin (1 μg/ml). Culture supernatants were assayed for IL-2 and IFN-γ after 18 h. (**D**) The four successive washes described above in **C** were analyzed by SDS-PAGE and immunoblotted with anti-lactoferrin as described in legend of Fig 5a. Representative of 1 out 3 experiments is shown. (**E**) IL-2 and IFN-γ secretion from the culture supernatants (as described above in **C**) are shown as bar graphs. Data representative of the average of 3 independent experiments are presented. The error bars indicate the standard error of the mean between triplicates.

Next, spleen cells were stimulated with SEA in the presence of lactoferrin to determine its impact on cytokine synthesis. Lactoferrin strongly inhibited IL-2 production while control BSA did not (Fig 6a, top panel). Transferrin was also detected by mass spectrometry in BALF fractions (Table 1), and inhibited IL-2 release. In contrast to stimulation with SEA, cells stimulated with anti-CD3/-CD28 in the presence of lactoferrin, and not transferrin, inhibited IL-2 production (Fig 6a, bottom panel). Thus, IL-2 synthesis from naïve T cells was inhibited by lactoferrin under TCR specific and mitogenic stimulation that also included PMA + Ionomycin (Fig 6b). Effector T cells generated by immunizing mice with SEA + LPS [26,27] were slightly inhibited by lactoferrin, although not to statistical significance (Fig 6b). To assess if transient lactoferrin treatment could mediate long term inhibition of IL-2 production, cells were pre-incubated with lactoferrin or BSA for 3 h and washed before overnight stimulation with SEA or PMA + Ionomycin (Fig 6c). Lactoferrin was washed away as determined immunoblot (Fig 6d). After overnight stimulation comparable IL-2 levels from lactoferrin and BSA treated cells were observed (Fig 6e), demonstrating that continuous presence of lactoferrin was required.

### Lactoferrin causes T cell death and blocks early cytokine synthesis

**Fig 7:**
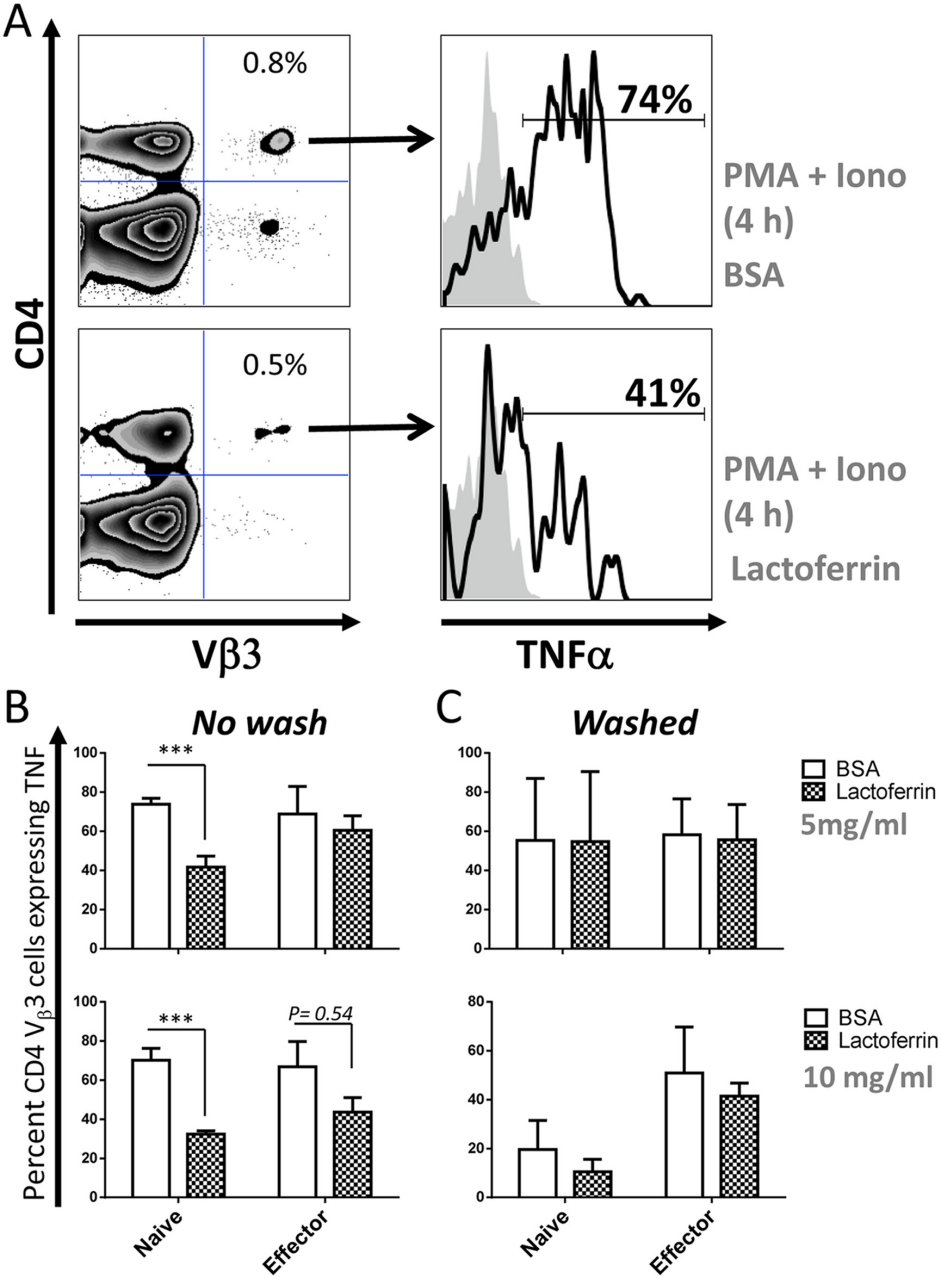
Lactoferrin significantly inhibits early intracellular TNF synthesis in naïve over that made by effector T cells. (**A**) Splenocytes from naïve mice were incubated with lactoferrin or BSA (5 mg/ml), in the presence of BFA and stimulated with or without PMA + Ionomycin for 4 h at 37°C. Cells were stained for CD4 and TCR Vβ3, fixed, permeabilized and stained for intracellular TNF. Representative dot plots show the gating strategy. The percentage of SEA-specific T cells: CD4^+^Vβ3^+^, is indicated in top right quadrant (left panels) and the percentage of those cells producing TNF is indicated as histograms in the right panels. (**B**) The mean percentage +/- standard error of the mean of CD4^+^Vβ3^+^ cells producing TNF from 3 independent biological replicates was analyzed as described in **A** is shown. Hence, naïve and effector splenocytes were obtained as described in the legend of Fig 6. One set of cells were directly incubated with lactoferrin or BSA in the presence of BFA and PMA + Ionomycin (*No wash*, left panels) and stained as described in **A**. Another set of cells (*Washed*, right panels) was first incubated for 3 h with lactoferrin or BSA, washed 4 times as described in legend of Fig 6c and then stimulated with or without PMA + Ionomycin for 4 h at 37°C. Data are combined from 3 experiments with n = 3 and displayed as mean +/- standard error of the mean. Statistical significance between BSA and lactoferrin was evaluated by two-tailed Student’s *t* tests. * p<0.05, ** p<0.01, *** p<0.001.

Based on these data we hypothesized that lactoferrin-mediated inhibition might be the result of decreased cell survival. To test this, the cells were stained with a marker of mitochondrial integrity after overnight stimulation as described in Fig 6a and 6b. After incubation with lactoferrin the cells had decreased mitochondrial integrity compared to BSA (Table 2), suggesting reduced cell viability. However, cells washed after 3 h incubation with lactoferrin or BSA had comparable levels of mitochondrial integrity (Table 3). Thus, overnight incubation with lactoferrin was required to reduce IL-2 production likely by inducing cell death. To address if lactoferrin could perturb early cytokine production before affecting cell viability, intracellular cytokine staining of TNF after 4 h of stimulation with PMA + Ionomycin was used. SEA-specific T cells were stained with anti-CD4, -Vβ3 and -TNF (Fig 7a). Incubation with lactoferrin inhibited TNF synthesis in naïve T cells to a much greater extent than effector T cells (Fig 7b, left panels), however, increasing lactoferrin levels did begin to block TNF production (Fig 7b, bottom panel). Similar to IL-2 production (Fig 6), lactoferrin did not inhibit TNF production when it was washed away before stimulation with the mitogen (Fig 7b, right panels), demonstrating that cells exposed to lactoferrin are not irreversibly programmed to die. Thus, lactoferrin inhibits cytokine production more effectively in naïve than in effector T cells by causing cell death and blocking early cytokine synthesis.

**Table 2:** Overnight incubation with lactoferrin affects cell viability.

*Cells*:	Naive		Effector	
*Treatment*:	BSA	Lactoferrin	BSA	Lactoferrin
*Stimulation*:	**No stimulation** (percent viability)			
**Exp. #1**	47.8	0.4	35.5	0.9
**Exp. #2**	43.6	0.5	45.6	0.7
**Exp. #3**	40.2	37.8	41.2	32.9
Exp. #4*	40.6	0.7	34.2	0.8
*Stimulation*:	**SEA** (percent viability)			
**Exp. #1**	50.1	0.5	35.9	1.0
**Exp. #2**	40.2	0.2	38.2	0.4
**Exp. #3**	43.3	45.1	39.7	37.9
Exp. #4*	39.6	3.2	34.3	1.7
*Stimulation*:	**PMA + Ionomycin** (percent viability)			
**Exp. #1**	15.7	2.8	12.0	2.0
**Exp. #2**	12.8	3.7	13.3	1.1
**Exp. #3**	12.1	9.8	6.1	6.2
Exp. #4*	39.2	0.1	37.4	0.1

Cell were treated with proteins at 5 mg/ml, except Exp. #4* (10 mg/ml), stimulated for 18 h as described in legend of Fig 6a and 6b. Viability of cells was then assayed by flow cytometry using Mitoflow as a marker of mitochondrial integrity, the percentage of cell alive is reported in the table.

**Table 3:** 3 h incubation with lactoferrin does not affect cell viability.

*Cells*:	Naive		Effector	
*Treatment*:	BSA	Lactoferrin	BSA	Lactoferrin
*Stimulation*:	**No stimulation** (percent viability)			
**Exp. #1**	46.7	44.0	34.6	35.4
**Exp. #2**	52.5	51.8	38.2	36.0
**Exp. #3**	35.8	20.0	33.2	26.5
*Stimulation*:	**SEA** (percent viability)			
**Exp. #1**	50.9	50.1	38.3	40.7
**Exp. #2**	54.8	56.3	39.7	41.4
**Exp. #3**	42.9	21.8	30.3	18.9
*Stimulation*:	**PMA + Ionomycin** (percent viability)			
**Exp. #1**	10.5	10.8	5.2	6.5
**Exp. #2**	1.9	22.5	13.3	14.4
**Exp. #3**	14.2	15.7	10.0	10.3

Cell were treated with proteins (5 mg/ml), washed as described as in legend of Fig 6c, and stimulated for 18 h as described in legend of Fig 6c and 6e. Viability of cells was then assessed by flow cytometry using Mitoflow as a measurement of mitochondrial integrity, the percentage of cells alive is reported in the table.

## Discussion

Here it is shown that an exogenous pathogenic protein such as SEA can be detected outside of an active infection site using a functional bioassay. Moreover, we demonstrate a way mucosal immunity contains the powerful activity of superantigens. Firstly, trace levels of inhaled SEA were recovered from BALF as determined by mass spectrometry. This was evident even after the initiation of pulmonary inflammation and importantly the recovered SEA was bioactive showing the mucosal innate immune system could not entirely inactivate SEA even late after inhalation. Secondly, SEA fractionated with lactoferrin and transferrin demonstrating the potential of innate immune factors to shield adaptive immune cell stimulation from external agents. Lastly, lactoferrin inhibited SEA-mediated T cell cytokine production by enhancing cell death using a mechanism that relied on the continual presence of lactoferrin. Collectively, these results suggest that bioassays can add an important tool for detecting functional pathogen by-products in ways that molecular diagnostics cannot, and demonstrate that an innate barrier exists in the lung even prior to an inflammatory insult.

The bioactivity of BALF at 18 h after SEA intranasal instillation initially suggested the presence of a DAMP, alarmin, and/or cytokine with the capacity to stimulate IFN-γ release on IL-12 preconditioned cells. The activity was similar to IL-12 but less powerful than LPS or the alarmin IL-33 and resistant to boiling but sensitive to proteinase K (Fig 1a–1c). Nevertheless, the capacity to stimulate IL-2 supported the notion that SEA itself was recovered even though this was 16 h into the pulmonary inflammatory response (Fig 1d). In fact, the bioactivity of the BALF-SEA was fairly constant over a time course (data not shown), contrasting to previous data showing a 7-log reduction in viral particle recovery within 18 h of infection [28]. Several other attributes of superantigens suggested that recovery of SEA would be unlikely. The high affinity of SEA for MHC II is well documented [29]. Further, considering that alveolar macrophages and dendritic cells express MHC II [30], and SEA inhalation induces the rapid migration of monocytes to lung followed by upregulation of MHC II [17,31] it was reasoned that SEA would not be recoverable at later time points. Finally, the systemic nature of the SEA-induced response after inhalation is clear [6], which strongly suggests that SEA moves rapidly into the bloodstream. Nevertheless, BALF-SEA did not induce IFN-γ or IL-2 release in spleen cells devoid of T cells (Fig 2). Thus, this strongly suggested that SEA was in BALF and its bioactivity maintained. Using size exclusion chromatography it was demonstrated that SEA bioactivity was detected in fractions corresponding to a molecular weight between 15–45 kDa, and after subjecting these fractions to anion and cationic exchange a highly pure sample was recovered (Fig 4). After mass spectrometry it was clear that SEA was present. To our knowledge purification of bioactive SEA from BALF has not been previously reported, but interestingly only in trace amounts with about 9% peptide coverage (Table 1). In fact, immunoblotting for SEA yielded no detectable signal (Fig 5a). These results show the importance of a functional readout compared to detection alone.

While there is no doubt that detection is extremely useful in diagnosis and prediction outcomes, there is space for expanding the use of functional bioassays to better understand the effects of a presumed pathogen or factor. For instance, BALF analysis of ICU patients could include functional tests of fluid such as described here and elsewhere [32,33]. An example is the ARDS network established by the NIH which has gathered an impressive collection of BAL and respiratory fluid [34], among which some could be tested for enterotoxin bioactivity when *Staphylococcus aureus* is an established risk factor [35]. Although mouse cells were used here, human BALF containing enterotoxins should stimulate responses just as well since SEA is not species specific per se. Also, mouse cells devoid of T cells could be used as identity controls or cells from HLA-DR transgenic mice [36,37].

The presence of immunosuppressive factors in the BALF that co-fractionated with SEA was quite surprising given that this occurred well into the inflammatory response. One possibility is that the co-fractionation of lactoferrin, lysozyme and transferrin (Table 1) may be an innate barrier that is present even without the induction of immunity. In this study we focused on lactoferrin, an iron-binding glycoprotein with known immune effects [38,24], since it was detected with SEA (Fig 5b). Lactoferrin is found in mucosal secretions, synthesized by epithelial cells, present in neutrophilic granules [39] and in colostrum’s at concentrations up to 9 g/L [40]. Lactoferrin is a defensive factor in mice challenged with methicillin-resistant *Staphylococcus aureus* (MRSA) [38], and in cows infected with beta-lactamase resistant *Staphylococcus aureus* [41]. Moreover, lactoferrin has been shown to attenuate SEB activity [25], and clinically it has been used to prevent neonatal sepsis [42], and necrotizing enter colitis [43,40]. Thus, it is possible that these immunosuppressive factors can inhibit an environmental insult before an immune response is initiated. The data detecting lactoferrin in BALF from naïve mice is consistent with lactoferrin being part of a protective shield even in the absence of provocation. Nevertheless, since neutrophils release lactoferrin [44] it is also possible that it can be carried to sites of inflammation.

Of the two iron-binding proteins co-purified with SEA, lactoferrin had a broader spectrum of action compared to transferrin since it attenuated both anti-CD3/-CD28 and SEA stimulation while transferrin inhibited only SEA stimulation (Fig 6a). The reason for this difference is unclear, but may be related to the iron chelation capacity of transferrin [38]. Lactoferrin was more efficient at reducing IL-2 and TNF production from naïve than from effector cells. This could be linked to a differential expression of lactoferrin receptors after T cell activation [45]. Secondly, lactoferrin-mediated cytokine inhibition was in part due to a decrease of primary T cell survival (Table 2), possibly through a similar apoptotic mechanism as previously described in human T leukemia cells [46]. Lastly, a faster process occurred in the reduction of TNF at 4 h (Fig 7).

## Conclusions

In summary, our work suggests that detecting SEA bioactivity in BALF can be used as a functional clinical diagnostic, especially when molecular diagnostics fail. Two such cases might involve status asthmaticus [47] and undetected co-infections [11,48]. Importantly, the copurification of immunosuppressive proteins with SEA suggests that they could be applied as countermeasures against enterotoxin-mediated lung inflammation and injury.

## Abbreviations

BALF: bronchoalveolar lavage fluid
SEA: Staphylococcal enterotoxin A
DAMP: danger-associated molecular pattern
MRSA: methicillin-resistant *Staphylococcus aureus*
BSA: Bovine serum albumin
BFA: Brefeldin A
